# ASSERT (Acute Sacral inSufficiEncy fractuRe augmenTation) randomised controlled, feasibility in older people trial: a study protocol

**DOI:** 10.1136/bmjopen-2019-032111

**Published:** 2019-07-10

**Authors:** Dawn van Berkel, Terence Ong, Avril Drummond, Paul Hendrick, Paul Leighton, Matthew Jones, Khalid Salem, Nasir Quraishi, Cassandra Brookes, Ana Suazo Di Paola, Sarah Edwards, Opinder Sahota

**Affiliations:** 1 Health Care of the Older People Division, Nottingham University Hospitals NHS Trust, Nottingham, UK; 2 School of Health Sciences, University of Nottingham, Nottingham, UK; 3 Division of Physiotherapy and Rehabilitation Sciences, School of Health Sciences, University of Nottingham, Nottingham, UK; 4 Division of Primary Care, School of Medicine, University of Nottingham, Nottingham, UK; 5 Centre for Spinal Studies and Surgery, Nottingham University Hospitals NHS Trust, Nottingham, UK; 6 Leicester Clinical Trials Unit, University of Leicester, Leicester, UK

**Keywords:** geriatric medicine, spine, adult orthopaedics, qualitative research, health economics

## Abstract

**Introduction:**

Pelvic fragility fractures (PFF) are common in older people and associated with a significant burden of mortality and morbidity. This is related to the challenges of appropriate pain control and early mobilisation. The current standard for treatment of PFF is non-surgical management. Minimally invasive surgical techniques for sacral fracture stabilisation have been shown to improve outcomes in terms of pain control and mobility, and they are safe. Randomised controlled trials are required before recommendations can be made for surgical management of PFF to become the new standard of care. This feasibility study will explore several uncertainties around conducting such a trial.

**Methods and analysis:**

ASSERT (Acute Sacral inSufficiEncy fractuRe augmenTation) is a single-site randomised controlled, parallel-arm, feasibility trial of surgical stabilisation versus non-surgical management of acute sacral fragility fractures in people aged 70 years and over. Patients will be randomised to either surgical or non-surgical group on a 1:1 ratio. Follow-up of participants will occur at 2, 4 and 12 weeks with safety data collected at 52 weeks. Primary objectives are to determine feasibility and design of a future trial, including outcomes on recruitment, adherence to randomisation and safety. This will be supplemented with a qualitative interview study of participants and clinicians. Secondary objectives will inform study design procedures to determine clinical and economic outcomes between groups, including scored questionnaires, analgesia requirements, resource use and quality of life data. Data analysis will be largely descriptive to inform outcomes and future sample size.

**Ethics and dissemination:**

Ethical approval was granted by the North East Newcastle and North Tyneside 2 Research Ethics Committee (reference 18/NE/0212). ASSERT was approved and sponsored by Nottingham University Hospitals NHS Trust (reference 18HC001) and the Health Research Authority (reference IRAS 232791). Recruitment is ongoing. Results will be presented at relevant conferences and submitted to appropriate journals on study completion.

**Trial registration number:**

ISRCTN16719542; Pre-results.

**Strengths and limitations of this study**

- Descriptive analysis on effectiveness of outcomes will inform hypothesis testing in a future definitive trial, including levels of variability in order to power the trial appropriately.
- Nested semistructured interview study will provide valuable qualitative data to inform future definitive trial acceptability and processes.
- Determines the feasibility of economic measures including detailed resource use collection and quality of life data within the two arms, to aid the design of more comprehensive economic evaluation in a future definitive trial.
- The intervention is a proven safe surgical intervention, already used in existing healthcare practice, but further safety data in this cohort of patients will also be collected.
- A pragmatic trial set in an existing healthcare setting that may lead to a number of limitations on trial processes, including recruitment, adherence to randomisation and ease of data collection.

## Introduction

Pelvic fragility fractures (PFF) are common in older people, as they are a frequent presentation of osteoporosis, a condition characterised by low bone mass and structural deterioration of bone tissue, leading to bone fragility.^1^ Thus, PFF can occur as a result of low-energy trauma, typically following a fall from standing height or less.^2^ The reported overall incidence of PFF is variable, between 25 and 92 per 100 000 persons-years, with the highest frequency reported in females over the age of 75 years.^3–8^ Epidemiology studies worldwide have consistently shown a sustained increase in the age-adjusted incidence of PFF, with numbers expected to continue to rise exponentially over the next 10 years.^3 4 6 7 9–11^ These patients are also increasingly requiring inpatient admission for management of their PFF, representing a considerable ongoing burden to hospital services.^3 4 6 12^

The pelvis is a complex ring like structure composed of three principal bones; the paired innominate bones and the sacrum. Fractures of the pelvis are a heterogeneous group of fractures and are most commonly described by the Young-Burgess classification, which relates to the predominant direction of the vector force at the time of injury.^13 14^ Within this, lateral compression (LC) fractures are the most common and are further subtyped based on the resulting degree of displacement of the pelvic ring:^13^

- Type I: Oblique or transverse ramus fracture and ipsilateral sacral compression fracture.
- Type II: Rami fracture and ipsilateral posterior ilium fracture dislocation (crescent fracture).
- Type III: Ipsilateral LC and contralateral anterior-posterior compression (windswept pelvis).

The most commonly identified PFF presenting to hospital is that of the anterior ring in the form of fractures of the pubic rami.^12 15 16^ 60%to 90% of these patients will also have a concomitant posterior ring fracture in the form of an insufficiency fracture of the sacrum.^2 17–19^ Type 1 LC is therefore the most common subtype of PFF.^17 20^ While anterior pelvic ring fractures can be identified on plain X-ray, those fractures of the posterior pelvic ring are most typically identified on CT or MRI, which now has a much wider availability on emergency admission to hospital.^17 19 21^ From a biomechanical point of view, an undisplaced anterior pelvic ring is more stable than a posterior pelvic ring fracture, with the posterior ring providing the majority of structure and stabilisation of the pelvis on load-bearing.^2^

PFF, especially those involving the sacrum within the load-bearing posterior pelvic ring, result in pain-related immobilisation and increased care dependency.^21–23^ PFF have been shown to confer poor outcomes like those reported extensively in hip fractures but covets much less attention.^24^ Inpatient 30-day mortality sits at up to 11%, with a 12-month mortality up to 27%.^5 9 12 15–17 20 24–26^ This may be related to the demographics of patients admitted to hospital with PFF. This patient group commonly has significant comorbidities and over a third exhibit cognitive impairment, leaving them more suspectable to the medical complications of pain-dependant immobility and associated prolonged hospital stay.^5 17 25^ Inpatient mortality is often attributed to exacerbation of pre-existing comorbidity.^12^ Around half of the patients admitted with PFF develop hospital and immobility related complications including pressure sores, infection, renal injury, venous thromboembolism and delirium.^9 16 17 25–28^ The majority are unable to return home at their baseline level of mobility or independence on discharge.^5 25 27^ In excess of this, those with confirmed combined anterior and posterior ring insufficiency fractures have hospital stays 2 weeks longer than those with isolated anterior ring fractures, higher complication rates, 30% more chance of losing previous independence and higher rates of institutionalisation.^17 20 22 29^

Current standard care for PFF is conservative, consisting of systemic analgesia and mobilisation as tolerated.^30^ As a response to the high level of associated morbidity, management of PFF needs to be targeted at good early pain control in order to allow early mobilisation, return of independence and discharge.^22 31^ Currently, standard pain management consists of the use of systemic analgesia, especially opioids, but pain control adequate to allow early mobilisation is difficult to achieve in this cohort.^23^ Barriers to adequate pain management in PFF can include under-reporting of symptoms due to cognitive impairment, susceptibility to side-effects of opioids in the elderly and undertreatment due to perceived prescriber fear of opioid side-effects.^32^

Development of minimally invasive surgical techniques targeting fractures of posterior ring sacral fractures may provide an alternative to improve adequate pain control in this significant subset of PFF.^21^ Minimally invasive keyhole surgery techniques involving percutaneous cement augmentation with or without trans-sacral screw are increasingly being performed in order to stabilise sacral fractures.^21 22 31^ For those patients who have failed to progress with conservative management, these procedures have been shown to reduce pain and the amount of analgesia required postoperatively.^30 33 34^ This in turn allows increased patient mobility with a quicker return to baseline function and shortened length of stay as well as having an established safety profile.^9 22 30 33–39^ However, there are no randomised controlled trials that compare efficacy of sacral fracture surgery compared with conservative management in the early stages of recovery.^21 22 33^

## Methods and analysis

### Aims

The aim of this study is to determine the feasibility and design of a future randomised controlled clinical trial to evaluate the clinical and cost effectiveness of keyhole spinal sacral fixation (cement augmentation±screw fixation) compared with current standard practice of non-surgical management in older people presenting in the early stages to hospital with a Type 1 LC PFF.

### Objectives

The feasibility and final design of a definitive trial will be determined by fulfilment of the objectives outlined below. These are to:

- Determine the number of patients who meet the eligibility criteria in addition to recruitment (including willingness to be randomised) and retention rates of randomisedpatients.
- Explore the adherence of clinicians to the randomisation of patients within the trial.
- To collect outcome measure data for the assessment of mobility, pain and quality of life (face to face and self-reported measures), for potential use in a future definitive trial; estimate the mean and SD of these quantitative measures for hypotheses testing purposes.
- Evaluate ease of access and availability of information from current primary and secondary care databases, to determine the most efficient way of measuring associated patient level resource use.
- Use a qualitative nested interview study to assess participants’ and clinicians’ views on trial acceptability and processes to inform the design and conduct of a future definitive trial.
- Evaluate long-term safety of the intervention.

### Study design

The primary study design is a parallel, two-arm randomised controlled feasibility trial with participants allocated to either surgical or non-surgical intervention on a 1:1 ratio. A preliminary economic evaluation and a qualitative nested interview study will also be embedded within the feasibility study.

Participants will be recruited from a single site, Queens Medical Centre (QMC), Nottingham University Hospitals NHS Trust (NUH), a university teaching hospital serving a population of 700 000 and offering a tertiary spinal surgical unit.

### Participants

Participants presenting to NUH with a Type 1 LC PFF who fulfil the eligibility criteria, outlined below, will be approached for possible recruitment into the study. A fragility fracture is defined as a fracture sustained after low level trauma, usually a fall from standing height or less.

#### Inclusion criteria

- Aged 70 years and over.
- Ambulatory with/without walking aids prior to injury.
- Injury sustained within 28 days of presenting to hospital.

#### Exclusion criteria

- Complex pelvic fractures (eg, fractures involving/or close to the hip joint) requiring urgent surgery or progressive weight bearing exercises.
- Pathological fracture in the context of known or unknown malignancy.
- Previous surgery of the pelvis with metal obstructing the planned paths of the iliosacral screws.
- Condition that precludes surgery or general/spinal anaesthesia.
- Bedbound prior injury.
- Receiving palliative care.
- Moribund on admission.

### Recruitment

All patients admitted with a Type 1 LC PFF as identified on imaging (CT or MRI) will be invited to participate. The research team will be notified of the potential participant and will confirm eligibility with their clinical care team. The process for obtaining participant informed consent will be in accordance with Good Clinical Practice guidance and will include consent for potential inclusion in the qualitative interview nested study.

An Abbreviated Mental Test (AMT) will be used as a screening tool for capacity assessment. If the admission AMT completed by the clinical team is documented as 5–6/10, then it will be repeated by the research team at the time of screening. A participant will be assumed to have capacity if their AMT≥7/10 at either point of assessment. An AMT<7/10 will prompt a capacity assessment based on the principles of the Mental Capacity Act 2005 in relation to research.

Relatives or carers of potential participants who are unable to provide consent independently, will be approached as the participants’ personal consultee. If there is more than one relative or carer willing to act as the patient’s consultee, then they must all agree on the decision for the participant to be included in the study.

For patients or consultees who decline to take part, they will be asked if they would be willing to share their reasons this. It will be made clear that this is in order to help us improve the design and acceptability of the study and there is no obligation to do this. The findings will be tabulated into the final results.

### Randomisation

Consented participants will be randomly allocated to either surgical intervention or conservative non-surgical care on the day they consent via a secure web-based system (Sealed Envelope Ltd) by a member of the research team, ensuring allocation concealment. In order to minimise bias, participant baseline enrolment data will be entered into the randomisation system to be stratified prior to intervention allocation. Randomisation to the intervention groups will be on a 1:1 basis.

### Interventions

*Intervention group* will receive surgical intervention by key-hole spinal sacral fixation as determined by the treating spinal surgeon based on the participant’s general condition, morphology of the fracture and surgeon’s experience. The surgery will be completed within 7 days of randomisation. Cement augmentation of the sacral ala will be undertaken in participants with unilateral or bilateral sacral fractures with minimal cortical comminution. Additional sacroiliac screw fixation will be offered to participants with extensive fracture patterns which affect both sacral ala with significant cortical comminution. Usual postoperative care, monitoring and rehabilitation will follow.

*Control group* will receive usual hospital care. Participants will be treated with appropriate analgesia and have regular input from the ward therapy team. Participants may be referred for surgical intervention if it is indicated by their clinical team. This will be recorded and data collected and followed up with intention to treat.

### Outcomes

The study procedures undertaken are directly related to the outcomes used in order to address the objectives of this feasibility study.

### Feasibility study outcomes

Primary outcomes:

- Number of eligible patients.
- Number of patients willing to be randomised and adherence to randomisation.
- Number of clinicians willing to randomise and adherence to randomisation.

Secondary outcomes:

- Rate of participant recruitment and retention.
- Data on the completeness and variability of proposed definitive trial outcome measures.
- Failure of non-surgical conservative care and adverse events (AEs) in both arms.

### Outcomes measures for the subsequent definitive trial

Primary outcome measures:

- Timed Up and Go test (TUG)^40^ as a measure of mobility requiring both static and dynamic balance.
- Roland Morris Disability Questionnaire (RMDQ)^41^ as a self-rated measure of physical disability caused by low back pain.

Secondary outcome measures:

- Abbreviated Mental Test (AMT) as an assessment of cognition.^42^
- Montreal Cognitive Assessment (MoCA)^43^ as an assessment of cognition.
- Functional Independence Measure (FIM)^44^ as a measure of disability severity.
- Clinical Frailty Scale (CFS)^45^ as an assessment of frailty.
- Charlson Co-morbidity Index^46^ as a prediction of 1-year mortality based on comorbid conditions.
- Numeric 0–10 Pain Rating Scale^47^ as a measure of average pain on mobilising.
- EuroQol 5 Dimensions (EQ-5D-3L) Score^48^ as an assessment of quality of life.
- Barthel Activities of Daily Living (ADL) Index^49^ as an assessment of care dependency.
- Fracture details/classification.
- Analgesia requirements.
- Surgery details.
- Health and Social Care resource use.
- AEs and readmissions (as part of the long-term safety review).

### Analgesia requirement

Analgesia requirement will be recorded as follows: each medication will be classified as a strong opioid (including oxycodone, morphine, fentanyl, pethidine, hydromorphone, buprenorphine and tramadol), mild opioid (including medications containing codeine or dextropropoxyphene) or non-opioid medications (including paracetamol and non-steroidal anti-inflammatory drugs). The participant will be given a score of 0, 1 or 2 in each of these three categories depending on the number of concurrent different medications being taken within each category. Opioid medication will also include a calculation of the oral Morphine Equivalent Daily Dose using the Opioid Dose Equivalence score.^50^

### Study procedures

**Figure 1:**
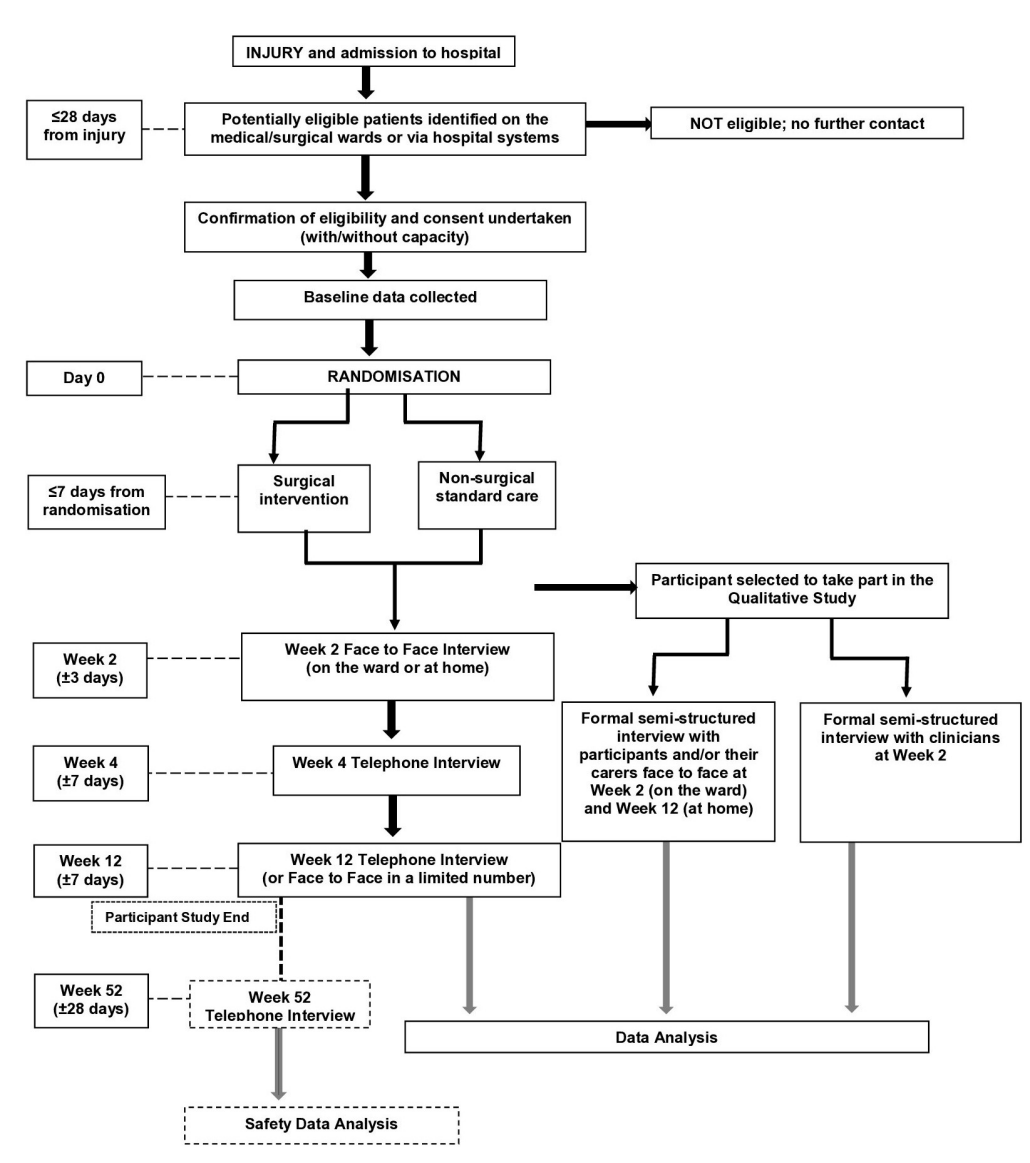
Participant flow through the trial including timings of data collection.

Participant flow through the trial is summarised in figure 1. Face to face contact with participants and/or carer will be required at baseline (considered day 0), week 2 and a limited number of participants at week 12. Telephone interviews will be conducted with participants at week 4 and for the majority of participants at week 12. Week 12 marks end of trial for the participant, with further contact made at week 52 as part of the long-term safety analysis.

**Figure 2:**
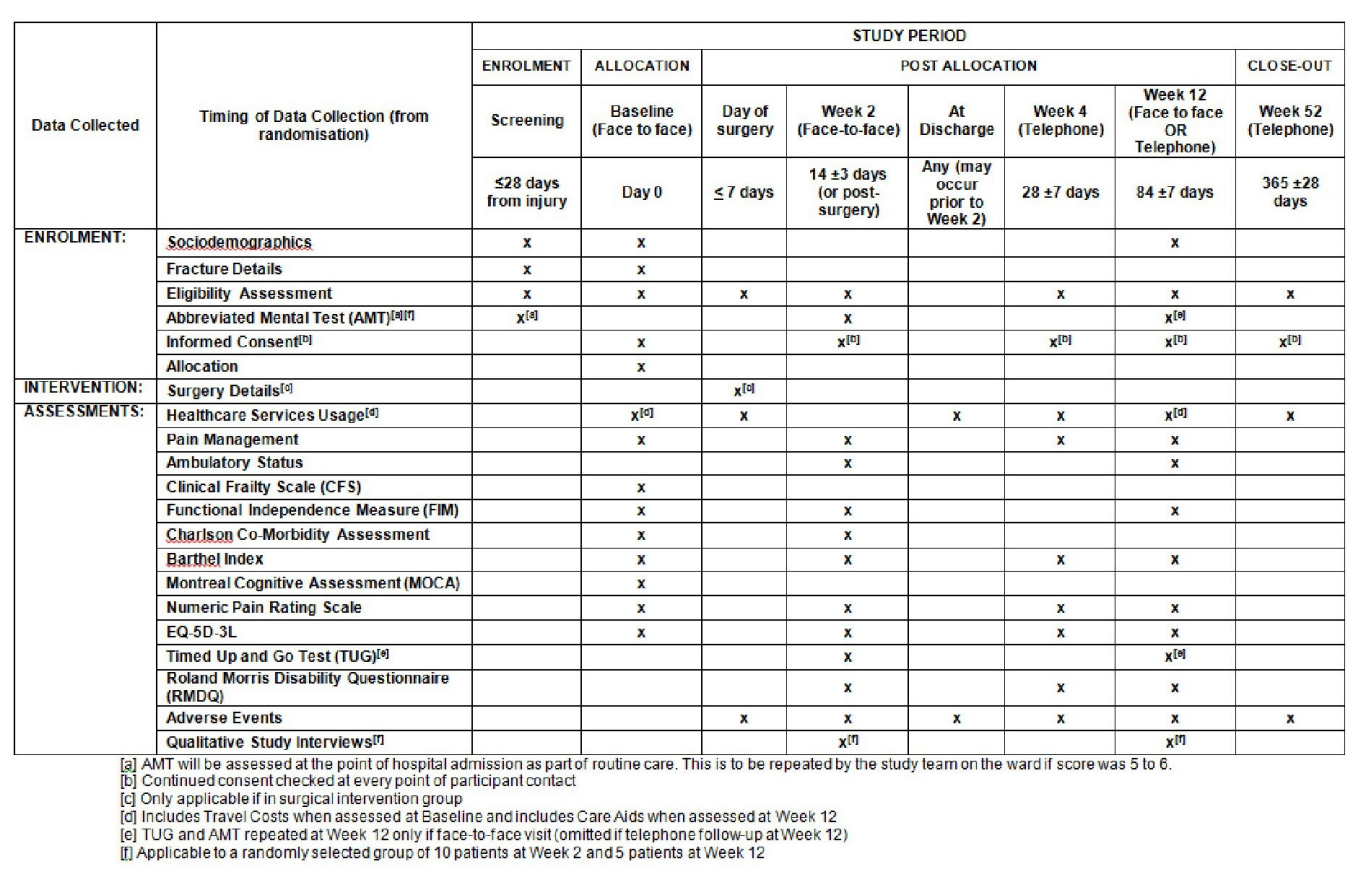
Schedule of enrolment, interventions and assessments.

Figure 2 shows the schedule of data collection, outlining which study procedures will be undertaken at what time point in the study period, measured from the point of randomisation. In addition, follow-up data at each time point will include participant still living, hospital length of stay, unplanned hospital readmission (within the first 28 days and 91 days post discharge) and all AEs, including surgical complications. For those participants that lack capacity, only clinically assessed questionnaires will be used. Participant contact will be conducted in the location the participant is residing at the time of the respective follow-up.

### Economic evaluation

Information about a participant’s treatment (including recorded resource use of the surgical procedure if applicable), hospital stay, emergency department, outpatient, readmission and primary care attendances (if related to ongoing management of the fracture) and social care needs will be gathered through discussion with participants, as well as hospital and primary care databases. An assessment of total resource use will be made at baseline, week 12 and week 52 in order to inform an economic analysis between the two treatment groups.

Individual prices of these health resources will be based on information from national tariffs, such as the Unit Costs of Health and Social Care^51^ for primary care resources, NHS Reference Costs^52^ for secondary care resources and the British National Formulary (BNF)^53^ for prescriptions. If the price for a resource cannot be found from the references above, a suitable estimate will be identified from consultation with the hospital finance department. Prices will be estimated at 2018–2018 prices.

### Qualitative assessment

Using maximum variation sampling, up to 10 participants will be chosen to undertake a semistructured face-to-face interview 7–10 days after randomisation. An interview topic guide will explore their views on the trial and recruitment process, the presentation of study information, study documentation and reasons for agreeing to randomisation. A smaller selection of five participants who complete the trial will have another shorter follow-up interview at week 12. The aim will be to further explore their experience of the trial, data collection processes and overall perception of participating. Further specific consent for this qualitative interview nested study will be taken in addition to that agreed at the point of trial recruitment.

A number of clinicians will also be asked to partake in a semistructured interview to explore their experiences of the study. These interviews will consider participant recruitment (eligibility and randomisation) as well as the process of integrating the research with the clinical team. All participating clinicians will complete informed consent for interview, recording and transcription.

### Sample size calculation

This feasibility study will aim to provide estimates of recruitment and retention rates, and the variability of important outcomes, in order to generate appropriate power calculations for the definitive trial. It is estimated that sample sizes between 24 and 50 are required for a feasibility study.^54–57^ Therefore, we propose to recruit for a 10-month period, from which we expect to screen approximately 100 patients. Our estimates are based on data from Gateshead Health Foundation Trust, who screened 67 patients with a similar eligibility criterion over a 12-month period within a smaller acute trust catchment population.^17^ We assume in this feasibility study that 20% of patients screened are not eligible and a 60% recruitment rate, so we expect to recruit 48 participants. By recruiting 48 patients, the estimated recruitment rate has a SE of 5.5% (95% CI 48.4% to 70.8%). Given the short active follow-up period, we are allowing for a lower 10% 3-month attrition rate. This estimates that 43 participants will complete the study, thus estimating the 90% retention rate with a SE of 4.4% (95% CI 77.3%; 96.5%). Completed follow-up on 43 patients will allow an estimated SE for the TUG of 1.2 s assuming the SD is about 8 s (95% CI 6.6 to 10.2) and an SE of 0.9 for the RMDQ, assuming the SD is about 6 (95% CI 4.9 to 7.6).

### Data analysis

Data analysis will primarily be descriptive to address the aims of the feasibility study. A statistical analysis plan will be agreed prior to database lock and a CONSORT flow diagram produced. Data analysis overall will inform future trial feasibility and the hypothesis analysis plan for a definitive trial.

Characteristics of participants recruited will be summarised using appropriate descriptive statistics and compared with patients who were eligible but not randomised. Completeness of data collection will be reported by intervention group and overall.

Descriptive summaries of outcome data at each follow-up time point will be presented by intervention group and overall. Outcome distributions for suggested floor and ceiling effects will be checked. Confidence intervals will be presented for the proportion of patients consented, randomised and retained in the trial completing assessment at 12 weeks, both overall and by treatment group. Confidence intervals for the SD of the secondary outcomes will also be calculated where appropriate.

Exploratory analysis of continuous outcomes for the subsequent definitive trial will be performed to investigate potential treatment effects. Differences in mean values between baseline and 12 weeks will be presented, with 95% CI. This feasibility trial is not powered to perform hypotheses testing; however, descriptive statistics of the difference between randomised groups will inform the design of the main definitive trial. No subgroup analyses are planned, and no interim analyses will be performed aside from routine checks of safety data.

### Health economic analysis

The within-trial economic evaluation will determine the cost-effectiveness of the surgical intervention compared with non-surgical (standard) treatment from an NHS and Personal Social Services perspective. The evaluation will follow the reference case guidance for technology appraisals as set out by NICE.^58^ Effectiveness will be captured using quality adjusted life years (QALYs) as assessed by the EQ-5D-3L.^48^ The primary outcome of the evaluation will be the incremental cost-effectiveness ratio (ICER) per additional QALY (ICER) gained from surgical fixation compared with standard care. Sensitivity analyses will be performed to control for uncertainty, which will include one-way and two-way sensitivity analyses on (but not exclusively) age, gender and baseline scores, with a probabilistic sensitivity analysis to control for all uncertainty. Results of the sensitivity analyses will be presented as tornado plots, 95% CI for the ICER and cost-effectiveness acceptability curves.

### Qualitative analysis

Qualitative interview data will be handled using the NVivo 12 software package and analysed using a framework approach informed by the literature about the challenges of clinical trial methodlogy.^59–63^ Initial thematic tables are likely to include elements such as randomisation and outcome measures. Table summaries will be used to generate recommendations about the nature and form of the subsequent trial; specific detail will also be used to inform recruitment strategies, data collection regimes and participant information resources.

### Data management and monitoring

Electronic data records will be stored in a SQL Server database, stored on a restricted access, secure server maintained by the University of Leicester, with access permission allocated by the Leicester Clinical Trials Unit (LCTU) IT team. Data monitoring for quality and completeness, including source data verification on a sample of documents, will be conducted by LCTU staff. The study documents shall be archived at secure archive facilities subcontracted to NUH. Data will be stored for 5 years.

Given this is a feasibility trial, the Data Monitoring Committee is included as part of the majority independent Trial Steering Committee (TSC), comprising two clinical experts and a statistician. The TSC will review trial progress, addressing study-related problems, assessing the safety of participants and ensuring timely publication of the study findings.

### Harms

All AEs will be reviewed by the chief investigator (CI) and recorded as part of the study outcome measures with an assessment of severity, relation and expectation. All deaths occurring up to the final study visit and serious AEs, other than expected surgical complications, will be recorded on the Sponsor SAE Form and faxed/emailed to the Sponsor and LCTU within 3 days of a researcher becoming aware of the event. Those related to the study and unexpected will be reported to the REC within 15 days. Events will be followed up until resolved or a final outcome has been reached.

The intervention in this trial is not testing a new surgical treatment. Therefore, serious expected sacroplasty surgical complications including wound infection, cement leakage causing nerve root damage and rarely pulmonary embolus will be captured in the Case Report Form (CRF), but do not require expedited reporting.

## Ethics and dissemination

### Patient and public involvement

Two members of the Royal Osteoporosis Society’s Nottingham support group represent the Patient and Public Involvement (PPI) for this study. Two focus groups have been held to inform the research, design and specific study outcomes. The PPI representatives have provided input into the grant application, study design and reviewing all participant facing documents. They will continue to provide input into trial conduct, as members of the Trial Management Group. They will assist with dissemination of study findings through their Royal Osteoporosis Society local communications as well as national contacts and support writing of the definitive future trial research grant application.

### Dissemination policy

Dissemination will include publication of the protocol methodology, with results being submitted for presentation at scientific meetings and conferences aimed at clinicians working with older people, trauma and spinal surgery (as well as being available on the NIHR RfPB website). Relevant patient groups and policy makers will be informed of the results, supported by our PPI engagement strategies.

If the findings indicate that a full-scale definitive trial is feasible, the data will be used to prepare an application for funding a large-scale definitive clinical and cost effectiveness randomised controlled trial, with the aim to change standard practise for the benefit of patient outcomes.

### Study registration and approvals

All study material has received approval from the Research Ethics Committee (REC—North East; Newcastle & North Tyneside 2, reference number 18/NE/0212), Health Research Authority (HRA) and the Nottingham Queens Medical Centre Research & Innovation department. Nottingham University Hospitals NHS Trust will act as sponsor to this study. The study has been registered on a clinical trials database (https://www.isrctn.com, reference number ISRCTN16719542, pre-results).

## Discussion

The growing older person population confers a large group of potential patients with complex medical and social needs, both in terms of medical comorbidities, susceptibility to hospital acquired complications and dependency. With the numbers of PFFs set to exponentially increase in the coming years, the potential healthcare resource burden within this group of patients is alarming. A recent systematic review concluded that randomised controlled trials were required to develop evidence-based protocols to reduce morbidity and mortality in older people with PFF.^22 33 64^ Given that keyhole spinal sacral fixation is already an established treatment option with a sound safety record, we propose that surgical management should be considered earlier in the treatment of PFF in older people admitted to hospital. This is to maximise early pain management with the aim of preventing pain-related immobilisation and its short and long-term consequences.

In order to ensure that the outcome of a clinical trial in this area has a high level of validity, it must be delivered within the constraints of the existing healthcare service, where the burden of patient care falls. This feasibility trial, delivered within this existing healthcare service, will analyse the outcomes posed by some of these constraints, to ensure that a future definitive trial is able to answer the clinical question efficiently. The inclusion of an economic evaluation will also demonstrate whether surgical fixation offers value for money as well as clinical effectiveness, an important consideration for existing services.

Potential limitations of delivering a clinical trial of this kind within an active healthcare service include identification of the sacral fractures themselves. Any patient presenting with an anterior pelvic ring fracture would need to be referred for further imaging in order to identify sacral fractures and thus be considered within the eligibility criteria for this trial. However, as standard care for patients presenting with PFF is currently conservative care, clinicians may feel that further imaging would not change a patient’s treatment course and thus be an unnecessary expense. As an identified sacral fracture is a key requirement for the eligibility criteria, this clinician assessment may significantly affect recruitment.

The target cohort in question may also provide further recruitment barrier. Cognitive impairment is common (up to 67%) in older patients presenting to hospital with PFF.^5 17 25^ As these patients confer such a large proportion of the real world PFF cohort, it would severely affect the validity of the trial to exclude them. Therefore, we have included a consent process for those patients that lack capacity. Identification is by AMT as a surrogate marker of capacity, which is completed as part of the clinical assessment of all admitted patients and therefore does not add any unnecessary burden prior to recruitment. Patients without capacity are reliant on the presence of relatives or carers to act as personal consultees, which may add a logistics barrier and reduce the recruitment of this subset of participants. Participants with cognitive impairment that are recruited may also be less likely to complete data collection due to difficulty with engagement, introduce detection bias due to issues with recall and may be more likely to be lost to follow-up.

Even once randomised, our participants remain under the existing healthcare service’s care for the entirety of the trial and are therefore at risk of protocol deviations due to the pragmatic setting of the study. The final decision to receive any intervention remains the responsibility of the patient’s clinical teams. For participants in the surgical intervention group, the decision to operate remains with the surgical team and may be susceptible to influence from factors such as surgeon experience and preference, belief in the clinical equipoise and theatre availability. Participants in the non-surgical (standard care) group may still be reviewed for surgical intervention based on clinical need identified by their clinical team, as determined by current practice. In order to assess the effect of this limitation, quantification and analysis of adherence to randomisation is an important outcome of this feasibility study.

An area of confounding not specifically assessed in this feasibility trial is the possibility of variation in the usual care received by all participants in both groups. This is not set by the protocol and while minimised by using a single site setting, where staff are working from the same local guidelines, resources and practices, variation is likely inevitable due to the non-regimented workings of a real-world healthcare service. The effect of these innate differences could be further minimised by using analysis of variation in outcome measures from this feasibility trial in order to power a future definitive trial appropriately.

This study is not powered to test the hypotheses, but the data collected will be able to provide a descriptive analysis on effectiveness of outcomes in order to inform analysis in a future definitive trial. The key outcomes address questions posed by the possible limitations of conducting such a trial within an existing public health service, specifically to recruitment and adherence to randomisation. The future aim is that the feasibility trial will advise a valid and fully powered randomised controlled trial to test the hypothesis that surgical intervention in PFF is of clinical benefit to patients as well as being cost effective and safe.

### Trial status

The study has been open for recruitment since October 2018 at QMC, with a current total of 9 recruited patients, and is ongoing. Estimated study duration is 30 months for a completion date of March 2021.

## Supplementary Material

Reviewer comments

Author's manuscript
