# Supplementary material for: ASSERT (Acute Sacral inSufficiEncy fractuRe augmenTation) randomised controlled, feasibility in older people trial: a study protocol
Source: BMJ Open. 2019 Jul 10;9(7):e032111. doi: 10.1136/bmjopen-2019-032111 (PMC6624053; doi:10.1136/bmjopen-2019-032111)
Supplement: Reviewer comments [file bmjopen-2019-032111.reviewer_comments.pdf]

PEER REVIEW HISTORY

BMJ Open publishes all reviews undertaken for accepted manuscripts. Reviewers are asked to complete a checklist review form (**http://bmjopen.bmj.com/site/about/resources/checklist.pdf**) and are provided with free text boxes to elaborate on their assessment. These free text comments are reproduced below.

ARTICLE DETAILS

**TITLE (PROVISIONAL)**	The ASSERT (Acute Sacral inSufficiEncy fractuRe augmenTation) Randomised Controlled, Feasibility in Older People Trial: study protocol.
**AUTHORS**	van Berkel, Dawn; Ong, Terence; Drummond, Avril; Hendrick, Paul; Leighton, Paul; Jones, Matthew; Salem, Khalid; Quraishi, Nasir; Brookes, Cassandra; Suazo Di Paola, Ana; Edwards, Sarah; Sahota, Opinder

This article was not externally reviewed at BMJ Open. Protocols that have been independently assessed prior to submission to BMJ Open are usually fast-tracked to publication on the grounds that further substantial changes will not be possible. This independent assessment will usually be external, independent review for both a substantial grant award from a non-commercial or government funder and ethics approval.
